# 68-Gallium DOTATATE scanning in symptomatic patients with negative anatomic imaging but suspected neuroendocrine tumor

**DOI:** 10.2217/ije-2017-0005

**Published:** 2018-02-02

**Authors:** Jasmine Shell, Xavier M Keutgen, Corina Millo, Naris Nilubol, Dhaval Patel, Samira Sadowski, Myriem Boufraqech, Lily Yang, Roxanne Merkel, Christine Atallah, Peter Herscovitch, Electron Kebebew

**Affiliations:** 1 Endocrine Oncology Branch, National Cancer Institute, National Institutes of Health, Bethesda, MD 20892, USA; 2 Positron Emission Department, National Institutes of Health Clinical Center, Bethesda, MD 20892, USA

**Keywords:** 68-Gallium DOTATATE, biochemistry, carcinoid, neuroendocrine tumor, symptoms

## Abstract

**Aim:**

The study's aim was to determine the utility of 68-Gallium DOTATATE positron emission tomography (PET)-CT scanning in patients with carcinoid-like symptoms and negative anatomical imaging.

**Methods:**

Retrospective analysis of 22 of 196 patients with carcinoid-like symptoms and no evidence of primary neuroendocrine tumor (NET) based on anatomical imaging and endoscopy who underwent 68-Gallium DOTATATE PET-CT as part of a prospective clinical trial.

**Results:**

Of the biochemically positive patients (n = 11), 18% (n = 2) had additional evidence of NETs based on 68-Gallium DOTATATE PET-CT. Of the patients identified by 68-Gallium DOTATATE PET-CT, 50% (n = 1) had a treatment change and 100% showed symptom improvement. Of the biochemically negative patients (n = 11), 68-Gallium DOTATATE PET-CT identified NETs in 64% (n = 7). Change in management occurred in 71% patients, and 57% of patients showed symptom improvement.

**Conclusion:**

68-Gallium DOTATATE PET-CT imaging is useful in detecting NETs in symptomatic patients with negative anatomical imaging and changes the treatments in these patients.

Neuroendocrine tumors (NETs) originate from neuroendocrine cells. They may be found throughout the body and might present with a spectrum of symptoms due to their ability to produce hormones. NETs may be indolent or aggressive, nonfunctional or functional. The term carcinoid was first used in 1907 to describe these tumors because they were ‘cancer like’ [1]. NETs include carcinoids, gastrinomas, insulinomas, glucagonomas, VIPomas, somatostatinomas, medullary thyroid tumors, pituitary tumors, paragangliomas and pheochromocytomas. According to an analysis of the Surveillance, Epidemiology, and End Results (SEER) database, there has been an increased incidence of NETs since SEER's inception, from 1.09 in 100,000 persons in 1973 to 5.25 in 100,000 persons in 2004 [1]. Yao *et al*. studied the epidemiology and prognosis using the SEER database and found that 41% of NETs were in the foregut, 26% were in the midgut, 19% were in the hindgut and 13% had unknown primary sites [1].

There are different methods of classification of NETs. One commonly used system is the WHO grading system. The WHO system classifies NETs into categories-based proliferation index as measured by Ki67 staining and the mitotic rate. Grade 1 tumors are least aggressive, followed by grades 2 and 3 which are the most aggressive [2,3]. Treatment options are often dictated by whether the extent of disease (localized, regional or distant metastases), WHO tumor grade and functional status of the NET. Surgery is the only curative and preferred treatment in most localized NET with a malignant potential or if functional and causing metabolic complications [3]. Additional treatments used for low-grade NET that are not resectable include somatostatin analogs, everolimus and sunitinib [3]. Chemotherapy which includes alkylating agents such as streptozotocin and temozolomide, antimetabolites such as 5-fluorouracil and capecitabine and platinum derivatives is used for high-grade tumors, neuroendocrine carcinomas [3].

Common biochemical markers used to aid in the diagnosis of NET include chromogranin A, serotonin, 5-hydroxyindole acetic acid, pancreastatin, neuron-specific enolase, pancreatic polypeptide, neurokinin A as well as disease-specific biomarkers, such as gastrin and insulin [4].

Symptoms typically observed in patients with carcinoid tumors are flushing, diarrhea, constipation, palpitations, hypertension, tachycardia, shortness of breath and headache [5]. NETs occur in multiple inherited syndromes, including multiple endocrine neoplasia types 1 and 2 (MEN1, MEN2), von Hippel–Lindau syndrome, neurofibromatosis type 1 and tuberous sclerosis [1]. Some patients with suspected NETs can present with typical carcinoid-like symptoms initially or after curative resection, but have no biochemical or imaging evidence of disease. This situation is often challenging and frustrating to both patients and clinicians.

68-Gallium DOTA peptide (DOTATATE, DOTATOC and DOTANOC) positron emission tomography (PET)-CT is a type of functional imaging in which a radioisotope-labeled somatostatin analog peptide binds to the somatostatin receptor found in NETs, with high-affinity primarily binding to somatostatin receptors 2, and sometimes 5, depending on the peptide used. The advantage of fusion imaging with PET-CT is that it can provide anatomic detail [6]. 68-Gallium DOTATATE imaging in humans is safe with relatively little toxicity. The associated complications that have been observed in the literature include discomfort at the injection site, which is usually self-resolving, elevated glucose, especially in patients who are glucose intolerant, and one case of postscan tachycardia, which was also self-resolving [7]. The radiation-effective dose per scan, 1.1 Roentgen equivalent man (REM), is well below the yearly maximum of 5.0 REM [8]. Precautions which should be taken in the case of patients undergoing 68-Gallium DOTATATE PET-CT scan are due to the use of radioisotope and it should not be used during pregnancy, breastfeeding and in patients less than 18 years [9]. There are no significant toxicities of scanning dose use of 68-Gallium DOTATATE with regards to renal and hematological side effects. In several studies, 68-Gallium DOTA-peptide PET-CT has been found to be highly sensitive for detecting NETs, with 68-Gallium DOTATATE PET-CT being most accurate [10–12].

Other imaging modalities that have been used in the evaluation of NETs include CT scan with or without contrast and MRI for anatomic imaging modalities. In addition to 68-Gallium DOTATATE PET-CT, functional imaging modalities include ^111^ln-octreoscan, a long-acting somatostatin analog which has historically been used as an important imaging technique in cases of NETs [13]. 18-fluoro-D-glucose PET-CT scan may be used in case of high-grade NET [14]. In this study, we sought to determine the utility of 68-Gallium DOTATATE PET-CT in patients with carcinoid-like symptoms and negative anatomical imaging and endoscopic evaluation, with or without biochemical evidence of disease.

## Methods

### Patients & imaging studies

A total of 196 patients were enrolled in a prospective protocol between 2013 and 2016 (Evaluation of 68-Gallium-DOTATATE PET-CT for Detecting Primary and Metastatic Neuroendocrine Tumors; NCT01967537). The purpose of this study was to evaluate the accuracy of 68-Gallium DOTATATE PET-CT in detecting primary and metastatic NETs. The clinical protocol was based out of the National Institutes of Health Clinical Center. After written informed consent was obtained, patients underwent anatomic imaging with CT scan of the chest, abdomen and pelvis as well as MRI of the abdomen. During the same visit, patients underwent functional imaging with 68-Gallium DOTATATE PET-CT scan. The inclusion criteria included age of 18 years or older, suspicion of NET on axial imaging, symptoms associated with NETs and a current or past diagnosis of a NET based on pathology or imaging as well as biochemical evidence of neuroendocrine pathology. Biochemical evidence of disease was based upon biochemical testing, which included measuring fasting serum chromogranin A, pancreatic polypeptide, neuron-specific enolase, vasoactive intestinal polypeptide, gastrin, somatostatin, insulin, calcitonin and glucagon, 24-h urinary catecholamine and metanephrine and 5-hydroxyindoleacetic acid [8]. Pregnant or lactating women were excluded. Symptoms of NET experienced within our cohort included diarrhea, flushing, dyspnea, abdominal pain and hypertension. Totally, 5 mCi (range ± 10%) of 68-Gallium DOTATATE was injected intravenously, and PET-CT imaging was performed 60 min after injection. Patients were placed supine in a PET-CT scanner, and images from the skull to mid-thighs were obtained [10]. A noncontrast CT scan was also performed for attenuation and anatomical location [7]. All patients had a triphasic-contrast CT of the neck, chest, abdomen and pelvis. An MRI scan was obtained for patients with an allergy to intravenous contrast. All the patients in our cohort had ^111^ln-octreoscan because the initial primary objective of the clinical trial was to compare the performance of 68-Gallium-DOTATATE PET/CT to that of ^111^ln-octreoscans.

This study focuses on 22 of 196 patients who had a history of negative anatomical imaging but who had symptoms typical of functional NETs, such as flushing, diarrhea, shortness of breath, abdominal pain and labile blood pressure (Table 1). These patients were classified based on their biochemical testing results. We used the official radiologic interpretation of our nuclear medicine physician to determine whether there was any positive or negative finding on the 68-Gallium DOTATATE PET-CT scan. The radiologic interpretation of the 68-Gallium DOTATATE PET-CT scan was done by a board certified nuclear medicine physician. The results of all imaging were interpreted independently. Of note, there were some findings considered equivocal per report and awaiting pathologic confirmation following surgical intervention.

**Table 1.** **Clinical characteristics of study cohort.**

**Variable**	**Biochemically positive**	**Biochemically negative**	**Total cohort**
**Sex (n)**			

Male	6	0	6 (26%)

Female	5	11	16 (72%)

**Age (mean) ± SD**	49.36 ± 15.12	51.09 ± 9.76	50.23 ± 12.76

**Symptoms**			

Diarrhea	3	7	10 (44%)

Flushing	4	7	11 (48%)

Dyspnea	0	2	2 (9%)

Abdominal pain	2	2	4 (17%)

Hypertension	3	3	6 (26%)

**Previous history of NET completely removed**			

Negative	6	4	10 (45%)

Positive	5	7	12 (54%)

**Prior workup**			

Upper endoscopy	8	3	11 (50%)

Colonoscopy	5	4	9 (41%)

Endoscopic ultrasound	5	0	5 (21%)

Capsule endoscopy	1	0	1 (4%)

Bronchoscopy	0	1	1 (4%)

Treatment change based on 68-Gallium DOTATATE imaging showing sites of NETs	1	5	6 (27%)

**Interventions**			

Surgical resection	1	1	2 (9%)

Systemic therapy: ^111^ln-octreoscan, lanreotide, sunitinib or peptide receptor radionuclide therapy	10	10	20 (91%)

NET: Neuroendocrine tumor; SD: Standard deviation.

### Data analyses

All patients were seen annually for follow-up and contacted by phone to determine symptom response after intervention. 68-Gallium DOTATATE PET-CT results were compared with anatomical imaging (CT/MRI) and ^111^ln-octreoscan imaging results. Categorical data were analyzed using Fisher's exact test. A p-value of < 0.05 was considered significant.

## Results

Of the 22 patients, 10 did not have a history of pathologic diagnosis of NETs and 12 had a history of completely resected NETs. All 22 patients were symptomatic, and 11 of the 22 patients had elevated values in one or more of their biochemical testing results (Table 2). Symptom duration prior to diagnosis lasted an average of 39.75 months. The average follow-up was 13.5 months (range: 11–20 months). Of those patients who presented after already having a pathologic diagnosis, one was diagnosed based on a lymph node biopsy, two had biopsies that revealed gastric lesions, seven were diagnosed based on resections of obstructive or suspicious lesions and two were diagnosed after colonoscopic biopsy of colon and terminal ileum polyps. Of the resections, three were pancreatic and four were small bowel. These patients had known previous diagnoses and removal of NETs, with residual symptoms of unknown origin.

**Table 2.** **Biochemical testing values in study cohort.**

**Biochemical test**	**Percentage of patients with elevated values in total cohort (n = 22)**
Chromogranin A	35

Pancreatic polypeptide	0

Neuron-specific enolase	0

Vasoactive intestinal peptide	4

Urinary 5-HIAA	12

Gastrin	22

Urine-free metanephrine	4

Urine normetanephrine	9

Urine total metanephrine	4

Urine epinephrine	0

Urine norepinephrine	0

Urine dopamine	9

Somatostatin	0

5-HIAA: 5-hydroxyindoleacetic acid.

In seven of 11 patients (64%) who had no evidence of NETs based on biochemical testing, 68-Gallium DOTATATE PET-CT showed evidence of disease, six patients had metastases (85%, liver) and one had primary tumors (14%, duodenum; Table 1). Five of the seven patients (71%) had a change in treatment which included three patients who had a change in the medical management and two patients deciding on surgical resection, with one patient having histopathologic confirmation of NET. Four (57%) of the seven patients had symptom improvement at last follow-up.

Of the 11 patients who were symptomatic and had biochemical evidence of NETs with negative anatomic imaging, 68-Gallium DOTATATE PET-CT scans were positive in two patients (18%); one patient had locoregional disease (pancreas) and one patient had metastatic disease (lymph node). A total of 50% patients with a positive 68-Gallium DOTATATE PET-CT scan had a change in management and 100% of these patients showed symptom improvement regardless of whether or not they had a change in management.

**Figure 1.**
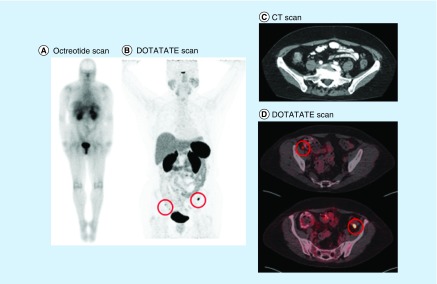
**Representative imaging in a 65-year-old female who presented to our institution status post-small bowel resection with pathology showing neuroendocrine tumor, grade 2.** She was considered free of disease at that time; however, she continued to have symptoms of diarrhea, flushing and fatigue. **(A)** Whole-body ^111^ln-octreoscan was negative. **(B)** Whole-body DOTATATE scan showing uptake in the right mesocolon and descending colon. **(C)** CT scan showing no positive findings (contrast and axial imaging). **(D)** DOTATATE scan showing uptake in right mesocolon and descending colon (axial imaging).

An example case is of a female patient in her sixth decade of life who presented to our institution with pathologically confirmed grade 2 NET of small bowel following a curative resection. She was considered free of disease at that time; however, she continued to have symptoms of diarrhea associated with abdominal pain, flushing and fatigue. We obtained a 68-Gallium DOTATATE scan which revealed positive uptake in the right mesocolon as well as descending colon, not previously noted on anatomical imaging. Both lesions were approximately 1.0 cm and, after discussing options with her, we recommended observation with medical management including Sandostatin LAR. On follow-up, she reported no complaints of diarrhea, fatigue or flushing (Figure 1).

68-Gallium DOTATATE PET-CT could detect significantly more lesions in patients with negative anatomical imaging compared with ^111^ln-octreoscan (30 vs 2; p = 0.028). The increased sensitivity seen in our cohort with use of the 68-Gallium DOTATATE PET-CT scan, has allowed us to discontinue regular use of the ^111^ln-octreoscan. Not only does the use of 68-Gallium DOTATATE PET-CT scan allow more comprehensive evaluation of our patients, but it also allows for a lower radiation dose per scan. The 22 patients in the current study had negative anatomical imaging based on outside imaging. Anatomic imaging which was repeated as part of our clinical protocol also did not show any lesions.

We did observe one false-positive case during the analysis of this cohort. The case is of a 44-year-old woman with no previous diagnosis of NET, who presented with symptoms of flushing, diarrhea and fatigue and had negative anatomical imaging and endoscopy. 68-Gallium DOTATATE detected lesions in the pancreatic tail and liver. After returning to her home institution, she underwent a distal pancreatectomy and wedge resection of segment IV of her liver. Pathology of the suspicious pancreatic lesion showed islet cell hyperplasia and pathology of the liver lesion was benign steatosis.

## Discussion

In this study, we found that 68-Gallium DOTATATE PET-CT is accurate for detecting initial or recurrent NETs in patients with carcinoid-like symptoms and negative anatomical imaging. 68-Gallium DOTATATE PET-CT could provide clinically relevant information in both biochemically positive and biochemically negative patients. Another important finding was that patients who had positive 68-Gallium DOTATATE PET-CT scans could receive appropriate intervention and showed symptom improvement.

We found that, compared with the ^111^ln-octreoscan, 68-Gallium DOTATATE PET-CT could detect significantly more lesions within this unique cohort of patients. Similar findings have also been reported in other studies. Pfeifer *et al*. reported a sensitivity of 87–88% for ^111^ln-octreoscan compared with 97% for 64-Cu DOTATATE PET-CT in a prospective analysis of 112 patients with pathologically confirmed NETs [15]. Srirajaskanthan *et al*. compared 68-Gallium DOTATATE PET-CT scans with ^111^ln-octreoscan scintigraphy in 51 patients with histologically confirmed NETs and found that 68-Gallium DOTATATE PET-CT scans detected 74.3% of the lesions, whereas ^111^ln-octreoscan detected 12% of the lesions [16].

Although many studies have evaluated the clinical utility of 68-Gallium DOTATATE PET-CT in NETs [10–11,16–19], this is the first study that shows an advantage to use 68-Gallium DOTATATE PET-CT scans in patients who were symptomatic, but had negative anatomic imaging and endoscopic evaluation, and in patients who had no biochemical evidence of disease. In one study, NET screening with 68-Gallium DOTATATE PET-CT in patients with MEN1, in symptomatic and asymptomatic patients, also detected that a high rate of NETs not seen in ^111^ln-octreoscan, anatomic imaging and endoscopy [10].

There are several limitations to our study. The sample size is small, but so, too, is the population of patients who have symptomatic but negative anatomic imaging and endoscopic evaluation, especially when considering patients who have no biochemical evidence of NETs. Although comprehensive biochemical testing was performed, several markers of NETs, such as pancreastatin and serotonin, were not measured in our cohort. There were 11 patients who had this testing at other institutions prior to enrolling in the clinical protocol. The markers were elevated in five of the 11 (45%) patients. There were patients with positive findings on 68-Gallium DOTATATE PET-CT scan who did not have operative intervention and thus no pathologic confirmation. Thus, we cannot exclude false-positive results. However, these patients had evidence of metastases on functional imaging not likely to be false-positives.

As of July 2016, the US FDA has approved the use of 68-Gallium DOTATATE for PET imaging for both adult and pediatric patients and the imaging modality has become widely available in the USA. The cost of the imaging modality is about USD$4,000.00 and this cost is not yet routinely covered by insurance companies.

In summary, we found that 68-Gallium DOTATATE PET-CT was helpful in detecting NETs in symptomatic patients who had no evidence of disease based on anatomical imaging and endoscopic evaluation, with or without biochemical evidence of disease. 68-Gallium DOTATATE PET-CT significantly altered treatment in these patients who, on follow-up, had improvement in their symptoms. We believe patients who have this type of difficult diagnosis should be offered 68-Gallium DOTATATE PET-CT.

**Summary points**

- 68-Gallium DOTATATE PET-CT is useful in patients with negative anatomic imaging and endoscopy studies who have carcinoid-like symptoms and or are biochemically positive for neuroendocrine tumors.
- 68-Gallium DOTATATE PET-CT identifies neuroendocrine tumors that impacts the management of patients who have negative anatomic imaging and endoscopy studies but have carcinoid-like symptoms and or are biochemically positive for neuroendocrine tumors.
